# Computational investigation of potential natural compounds as inhibitors of monkeypox virus cysteine proteinase

**DOI:** 10.3389/fbinf.2025.1637207

**Published:** 2025-07-28

**Authors:** Riya Nalwa, Anis Ahmad Chaudhary, Mandeep Chouhan, Prashant Kumar Tiwari, Saurabh Gupta, Hassan Ahmed Rudayni, Vivek Dhar Dwivedi, Sanjay Kumar

**Affiliations:** ^1^ Biological and Bio-computational Lab, Department of Life Science, Sharda School of Bio-Science and Technology, Sharda University, Greater Noida, Uttar Pradesh, India; ^2^ Department of Biology, College of Science, Imam Mohammad Ibn Saud Islamic University (IMSIU), Riyadh, Saudi Arabia; ^3^ Department of Biotechnology, GLA University, Mathura, Uttar Pradesh, India; ^4^ Center for Global Health Research, Saveetha Medical College and Hospitals, Saveetha Institute of Medical and Technical Sciences, Saveetha University, Chennai, India; ^5^ Bioinformatics Research Division, Quanta Calculus, Greater Noida, India; ^6^ DST-Facility, Sharda University, Greater Noida, Uttar Pradesh, India; ^7^ Centre of Excellence in Artificial Intelligence in Medicine, Imaging and Forensic, Sharda University, Greater Noida, India

**Keywords:** Monkeypox virus, cysteine proteinase, structure-based virtual screening, phytochemicals, molecular dynamics simulation

## Abstract

**Introduction:**

Monkeypox is a serious viral illness that is rarely seen but is spread from person to person and from animals to humans. Cysteine proteinase, an essential enzyme involved in the replication of the monkeypox virus (MPXV), is one of many possible therapeutic targets for MPXV. The primary function of this enzyme is to cleave the precursor polyprotein into the distinct proteins required for viral assembly. The aim was to develop potential drugs that can inhibit the proteinase and stop the spread of the MPXV.

**Methods:**

Virtual screening, molecular docking, molecular dynamics simulation, and free binding energy calculations were used to explore the potential of 569 phytochemicals from a variety of plants that could inhibit the proteinase of the MPXV.

**Results:**

The four compounds (Unii-CQ2F5O6yiy, lithospermic acid, kaempferol, and rhamnocitrin) with the best docking scores displayed docking score values ranging from −9.5 to −7.4 kcal/mol and were used for further analysis. Out of these, Unii-CQ2F5O6yiy exhibited a docking score of −9.5 kcal/mol, indicating the highest binding to the proteinase. Unii-CQ2F5O6yiy had the most stable and consistent root mean square deviation (RMSD) of <3 Å.

**Discussion:**

We identified the top four phytochemicals that exhibited better docking scores than the reference compound. The RMSDs of proteins in all the phytochemical complexes exhibited acceptable deviation except for lithospermic acid, whereas atoms of Unii-CQ2F5O6yiy and kaempferol in their docked complexes displayed less fluctuation than the reference compound (<5.4 Å).

**Conclusion:**

Unii-CQ2F5O6yiy could be used as a potential antiviral agent for the management of monkeypox virus. However, further experimental validation under *in vitro* and *in vivo* conditions is required to confirm its antiviral activity against MPXV.

## 1 Introduction

Monkeypox virus (MPXV) is a viral disease that specifically affects animals, including monkeys, rodents, and other mammals. It can be transmitted in humans and causes a viral disease known as monkeypox. MPXV disease was first identified in 1958 in a group of monkeys (Yadav et al., 2025; Pastula and Tyler, 2022) and is primarily found in the Central and West African regions, particularly in remote parts of the rainforest (Rani J., 2022). This is a zoonotic double-stranded DNA virus that belongs to the family Poxviridae and has similarities to the variola virus, which causes smallpox, and to the cowpox virus (CPXV) and vaccinia virus (VACV) (Okyay et al., 2022; Tiecco et al., 2022; Pakran et al., 2024). There are two main clades of MPXV. (a) The Congo basin clade has been observed in Central Africa, and (b) the West African clade, which is highly dangerous, is rapidly spreading in nature. Initially, the occurrence of MPXV was documented in Africa during the 1970s, touching the borders of the Democratic Republic of Congo (Sklenovská, 2020). Now, MPXV is a global problem. However, not much information related to this virus is available in terms of its manner of transmission, diagnosis, risks, and severity (Luo and Han, 2022).

There are several means of transmitting MPXV from animals to humans, which is a subject of great interest. Some animals, such as rodents, dogs, and non-human primates, can transmit this virus to humans (Li et al., 2023). MPXV initiates infection in animals through respiration. Later, animals infect other mammals via respiratory droplets (Maal-Bared et al., 2022; Beeson et al., 2023). It also spreads rapidly through vertical transmission from a mother to her offspring (Lum et al., 2022).

Various MPXV symptoms have been observed in humans, including headaches, muscle stiffness, and facial rashes (Luo and Han, 2022). The rash goes through various phases, starting with the formation of blisters filled with fluid, which eventually form a crust. In most cases, this disease lasts only for a few days, and sick individuals recover automatically. However, in some cases, this disease has been found with severe effects, predominantly in individuals with weakened immune systems (Sharma et al., 2022). MPXV infection is less common in humans but could become more dangerous when a person comes into contact with infected animals (Samaranayake and Anil, 2022).

Various therapeutic strategies have been implemented since the outbreak of MPXV. The FDA approved JYNNEOS as an emergency vaccine in 2022 (Meo et al., 2022). The process of vaccination has a delayed protective effect on the immune system. Hence, the use of antiviral medications would be more beneficial for the immediate relief of pre-infected humans. There is currently no information available on FDA-approved medications for the treatment of MPXV. During the outbreak of MPXV in 2022, the FDA-approved tecovirimat was the only medication utilized for the treatment of severely affected patients (Dubey et al., 2023). Other antiviral drugs, including cidofovir and brincidofovir (CMX001), are also being used to protect against MPXV (Rao et al., 2023). Tecovirimat targets F13, an essential protein that is involved in the formation of the extracellular virus envelope (Ali et al., 2023). Cidofovir and brincidofovir interfere with the viral DNA polymerase enzyme function, which inhibits DNA synthesis (Rabaan et al., 2023).

Poxvirus is a unique virus that replicates its own nucleic acid or genes in the host cytoplasm and utilizes the host machinery for transcription and translation. The proteins produced during the translation process interfere with the synthesis of host proteins, leading to alteration of the cellular environment of the host (Walsh et al., 2013; Herbert and Nag, 2016). Among these proteins, cysteine proteinase (I7L core proteinase) is crucial for various stages of the poxvirus life cycle (Zephyr et al., 2021). Cysteine proteinases help in uncoating the virus during entry and releasing the genome into the host’s cytoplasm. It has been shown that cysteine proteinases are involved in the processing and maturation of viral proteins by cleaving larger precursor proteins into smaller functional proteins during viral replication (Wang et al., 2023). This proteinase also interferes with the function of the host immune system by degrading several essential components of the immune system, including cytokines, chemokines, and proteins involved in the inflammatory response (Donnelly et al., 2011).

Due to their ability to perform all these essential functions, cysteine proteinases can be very promising therapeutic targets in the management of this virus. The inhibitors that inhibit proteases have shown promising results in certain other viral diseases, such as HIV. TTP-6171 was discovered as an inhibitor of I7L. Currently, fosdagrocorat and lixivaptan are identified as inhibitors for MPXV that target cysteine proteinase (Dubey et al., 2023). Alandijany et al. discovered two drugs, omadacycline and minocycline, after screening tetracycline antibiotics against MPXV cysteine proteinase (Alandijany et al., 2023). The A42R profilin-like protein of MPXV is a critical drug target, which is involved in cell development and motility. Virtual screening identified seven compounds (PubChem CID: 11371962, ZINC000000899909, ZINC000001632866, ZINC000015151344, ZINC000013378519, ZINC000000086470, and ZINC000095486204) from a library of 36,366 compounds from Traditional Chinese Medicine TCM, AfroDb, and PubChem databases with favorable binding affinities (−7.2 to −8.3 kcal/mol) compared to tecovirimat (−6.7 kcal/mol) (Ashley et al., 2024). Additionally, NSC 319990, NSC 196515, and NSC 376254 compounds were found to inhibit MPVX methyltransferase VP39 protein (Thai et al., 2024).

In this study, a comprehensive computational approach has been implemented to identify those compounds that effectively target MPXV proteinase. The structure of MPXV proteinase was not known previously, so a model was generated after employing molecular dynamics simulation, and the protein’s most likely structure was further improved. Many phytochemicals were screened against this model protein, and four phytochemicals were selected under 250 ns molecular dynamics to determine the binding affinity and stability of the hit compounds against MPXV proteinase. The dynamic characteristics of the protein–ligand complexes were investigated. This analysis involved studying parameters such as root mean square deviation (RMSD), root mean square fluctuation (RMSF), and molecular mechanics generalized Born surface area (MMGBSA).

## 2 Materials and methods

### 2.1 Modelling of protein structure

The three-dimensional (3D) protein structure of the viral core cysteine proteinase has not yet been solved experimentally. The primary sequence of the cysteine proteinase target protein in FASTA format was obtained using the gene accession number NP_536495.1 from the GenPept database (Benson et al., 2013). Using this sequence, its 3D structure was modeled via Alphafold Colab v2.1.0, which allows prediction of the 3D structure of protein involving a machine learning approach that integrates physical and biological information about the structure of the protein (Jumper et al., 2021). Using this approach, five protein models were generated, and the best model was validated by PROCHECK using the SAVESv6.0-structure validation server (Laskowski et al., 1993; Kawsar et al., 2022a). The model that showed higher residues in the most favorable region and fewer residues in generously and disallowed regions in the Ramachandran plot was selected among all the generated models (Amin et al., 2022). In order to further verify this structure, the molecular dynamics (MD) simulation was carried out to check the stability of the modeled protein (Dubey et al., 2023).

### 2.2 Molecular dynamics (MD) simulation of the modeled structure

The dynamic stability of modeled structure of protein was analyzed by MD simulation at 100 ns, which was carried out using an HP Z2 workstation running the Desmond-maestro 2020–4 on the Linux operating system (Bowers et al., 2006; Desmond Molecular Dynamics System, 2021; Maestro, 2021; Prime, 2021, D. E. Shaw Research, New York, NY, 2021.3. Maestro-Desmond Interoperability Tools, Schrödinger, New York, NY, 2021.3., n. d.). The preparation of protein was carried out using the protein preparation wizard module of the Schrödinger suite, and then an orthorhombic box (10 Å × 10 Å × 10 Å buffer) was created for simulation using the system building tool. The complete system was submerged in a water bath with Na^+1^/Cl^−1^ as counter ions and transferable intermolecular potential with a four-point (TIP4P) water model, with salt and ion placement being excluded at 20 Å around ligand-binding sites. By designating 0.002 ps time steps for anisotropic diagonal position scaling, the constant pressure was kept for the MD simulation. A 20 psi NPT reassembly at 1 atm pressure was applied as the temperature was gradually increased to 310 K. The optimized potential for the liquid simulations (OPLS)-2005 force field was used for MD analysis to maintain the compactness of the entire simulation system at 1 g/cm^3^. To get representative conformations of the system, the frames produced by each trajectory were clustered according to RMSD (Chouhan et al., 2023). The clustering technique is carried out via the Desmond trajectory clustering tool integrated into the Maestro package (Maestro, Schrödinger, LLC, New York, NY, 2021-3., n. d.). From the cluster, the middle structure with the greatest population was chosen. This structure was utilized for virtual screening and binding pocket prediction.

### 2.3 Binding grid box

The Computed Atlas of Surface Topography of proteins (CASTp) was utilized to predict the binding pocket of the viral core of cysteine proteinase. This server allows prediction of a binding cavity of a protein structure using topological and geometrical properties (Tian et al., 2018). The best pocket discovered by CASTp had an area of 457.392 Å^2^ and a volume of 557.529 Å^3^. A grid box was formed using binding pocket residues that was detected by CASTp with dimensions of 30 Å × 30 Å × 30 Å and centered at 19.69 Å, −35.57 Å, −27.67 Å in the x, y, and z axes.

### 2.4 Preparation of compound library

We selected five plants in our study that exhibit antiviral properties: *Melissa officinalis* (Lemon balm), *Ocimum tenuiflorum* (Tulsi), *Mentha arvensis* (wild mint), *Mentha piperita* (peppermint), and *Pogostemon cablin* (Patchouli). The 3D structures of phytochemicals of these selected plants were downloaded from IMPPAT in SDF format (Mohanraj et al., 2018; Vivek-Ananth et al., 2023). A list of 2,531 compounds associated with these plants was generated through the IMPPAT database, and among them, 1962 were found as duplicates (Supplementary Table S1). Therefore, only 569 remaining phytochemicals were combined, and a library was formed using Progenesis SDF studio (Alldritt et al., 2019; Amin et al., 2021). Following the preparation of a compound library, these compounds were screened using MTiOpenScreen (Labbé et al., 2015).

### 2.5 Structure-based virtual screening

The prepared library of 569 compounds was used for structure based virtual screening at the binding pocket of modeled viral core cysteine proteinase at the MTiOpenScreen web server using center 19.69 Å, −35.57 Å, and −27.67 Å and size 30 Å × 30 Å × 30 Å in the x, y, and z axes (Labbé et al., 2015).

### 2.6 Redocking

The top ten selected compounds were redocked in order to identify the most interacting residues in the active site of the modeled viral core cysteine proteinase MD simulation between the proteinase and possible phytochemical compounds. The simulation was conducted under default parameters by altering the 30 Å × 30 Å × 30 Å grid size along the three (X, Y, and Z) axes, containing all the crucial residues in the 19.69 Å, −35.57 Å, and −27.67 Å area to provide sufficient space for ligand conformations using the Chimera-AutoDock Vina plugin setup (Pettersen et al., 2004). The top four phytochemical candidates were selected on the basis of the highest negative docking score. Maestro 12.8 was used to generate the 3D and 2D interactions. The TTP-6171 inhibitor was used as a reference compound, and a similar docking approach was employed for the reference compound to compare the binding poses of various phytochemicals (Supplementary Figure S2). Following that, the top four phytochemical compounds with the greatest negative docking scores were taken into account for additional absorption, distribution, metabolism, and excretion (ADME) study using the SwissADME webserver (Daina et al., 2017; Rana et al., 2021).

### 2.7 Pharmacokinetic study

The SwissADME online tool was used to analyze pharmacokinetic information. The canonical SMILES of these compounds were extracted from PubMed (https://pubmed.ncbi.nlm.nih.gov/) and entered into the software, which returned the structures of the compounds as well as several data values used to assess their physicochemical properties, lipophilicity, water-solubility, pharmacokinetics, and medicinal chemistry (Supplementary Table S3) (Kawsar et al., 2022b). At the same time, radar charts based on physicochemical factors assess their respective oral bioavailability (Figure 3).

### 2.8 Protein–ligand molecular dynamics (MD) simulation

The top four docked complexes were chosen from the virtual screening and the docking procedure were subjected to a 250-ns MD simulation using the Desmond module of the Schrödinger suite (Jorgensen and Tirado-Rives, 1988; Desmond Molecular Dynamics System, 2021; Maestro, 2021; Prime, 2021, D. E. Shaw Research, New York, NY, 2021.3. Maestro-Desmond Interoperability Tools, Schrödinger, New York, NY, 2021.3., n. d.). The protein–ligand docked complexes were examined under the same circumstances used in the MD simulation of the modeled protein structure. The same protocol was followed for the reference complex cysteine proteinase-TTP-6171 so that the intermolecular interactions for the selected phytochemical compounds can be compared with the reference compound.

### 2.9 Binding free energy analysis

The docking scores of the top four selected proteinase-ligand complexes were assessed using the prime molecular mechanics/generalized Born surface area (MM/GBSA) module of the Schrödinger suite (Jacobson et al., 2002; 2004; Prime, Schrödinger, LLC, New York, NY, 2021-3., n. d.). To evaluate the stability of the complexes, average binding free energy was calculated on the retrieved poses from the whole 250-ns MD simulation trajectory using the OPLS-2005 force field and default parameters against the reference complex proteinase-TTP6171 (Jorgensen and Tirado-Rives, 1988; Harder et al., 2016).

Here, all of the ions and solvent molecules were removed from the obtained poses, and the net free binding energy (∆G) was determined using Equation 1.

$$\Delta G_{\text{Bind}}=\Delta G_{\text{complex}\left(\text{minimized}\right)}-\left(\Delta G_{\text{receptor}\left(\text{minimized}\right)}+\Delta G_{\text{ligand}\left(\text{minimized}\right)}\right)$$ (1)

Here, Δ*G*_Bind_ represents the binding free energy; the ∆*G*_complex_ exhibits the binding free energy of the complex; ∆*G*_Receptor_ and ∆*G*_Ligand_ indicate the energy for the receptor and ligand, respectively. The lower the binding free energy, the better the binding of the ligand in the protein–ligand complex.

## 3 Results and discussions

### 3.1 Validation of modeled protein structure

**FIGURE 1:**
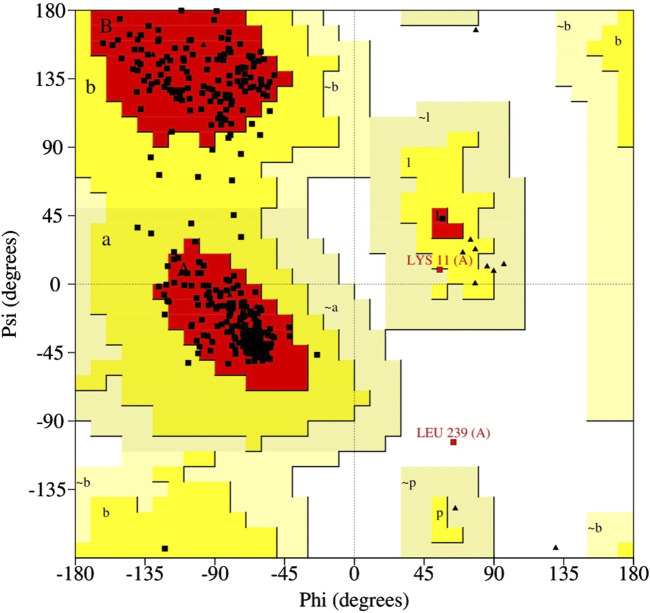
Ramachandran plot showing residues in favored, allowed, and disallowed regions.

For the modeled structure of cysteine proteinase in monkeypox virus, we found that 93.9% of the amino acid residues were in the favored regions, whereas only 0.3% of the amino acid residues were in generously allowed and disallowed regions of the Ramachandran plot (Figure 1). When the model with “higher residues in the most favourable region and fewer residues in generously and disallowed regions” was chosen, it indicated that the model had a more well-defined backbone conformation that closely resembled known protein structures, making it a more realistic representation of the actual protein’s structure. The RMSD of the modeled structure of cysteine proteinase in monkeypox virus showed insignificant deviation in the initial phase of MD simulation but attained a stable equilibrium (5 Å) over the course of the simulation (Supplementary Figure S1a). The root mean square fluctuations (RMSF) of the modeled cysteine proteinase showed <3.2 Å fluctuations throughout the simulation. However, it exhibited three peaks with RMSF >5.5 Å at the 50, 150, and 400 residual positions (Supplementary Figure S1b). This result showed that the protein structure is stable, and the stability of the protein suggests that the structure may be representative of the biologically relevant conformation.

### 3.2 Active site and binding pocket of proteinase

As previously reported, the “core” binding site motif (W-HW-Q-C) of the cysteine proteinase has five amino acid residues, whereas Cys328 and His241 form a catalytic motif in the active site of the cysteine proteinase. The Gln322 side chain produces a subtilisin-like oxyanion hole, in addition to the backbone amino group of Cys328. Trp242 and Trp168, two side chains, also help to create a narrow channel through which the substrate can enter (Dubey et al., 2023). The binding pocket contained the active site residue Cys328, as demonstrated by the CASTp result of the MPXV protein. Active site residues detected by CASTp were Met^136^, Asp^137^, Leu^138^, Lys^139^, Ile^140^, Arg^145^, Lys^160^, Glu^162^, Pro^163^, Tyr^238^, Leu^239^, His^241^, Lys^243^, Asp^258^, Gly^260^, Asn^262^, Ile^263^, Glu^266^, Asp^292^, Thr^294^, Asn^295^, Ile^298^, Val^320^, Gln^322^, Leu^323^, Leu^324^, and Glu^325^. The middle structure was extracted from the most populated cluster resulting from the 250-ns MD simulation and was further used for virtual screening against the phytochemical compounds collected from the IMPPAT database.

### 3.3 Virtual screening analysis

**FIGURE 2:**
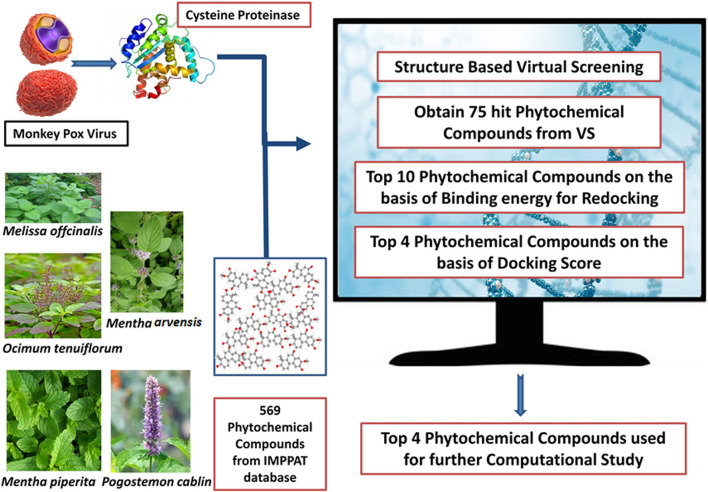
Workflow chart for the structure-based virtual screening process.

The virtual screening method was used to find small compounds with high affinity for the active pocket of the target protein cysteine proteinase from a large chemical database. The natural compound libraries are of great interest for screening due to their high chemical diversity, biochemical specificity, and other molecular descriptor properties that make them advantageous as lead ligands in drug discovery. Compounds of a phytochemical library were virtually screened against cysteine proteinase at the MTiOpenScreen webserver, which yielded 75 compounds with binding energy between −9.3 kcal/mol and −3.9 kcal/mol (Supplementary Table S2). The following top four phytochemical compounds, Unii-CQ2F5O6yiy, lithospermic acid, kaempferol, and rhamnocitrin, with docking scores between −9.3 kcal/mol and −7.4 kcal/mol, were then selected for further redocking and intermolecular interaction analysis (Table 1; Figure 2). They were compared with the reference compound, TTP-6171.

**TABLE 1:** List of top four selected phytochemical compounds virtually screened against the cysteine proteinase of *Monkeypox virus.*

S. No.	IMPAAT ID	Phytochemical name	Molecular formula	Phytochemical origin	Binding score	Redocking score
1	IMPHY004249	Unii-CQ2F5O6yiy	C_48_H_78_O_20_	*Ocimum tenuiflorum*	−9.3	−9.5
2	IMPHY006793	Lithospermic acid	C_27_H_22_O_12_	*Mentha piperita*	−8.8	−8.9
3	IMPHY004388	Kaempferol	C_15_H_10_O_6_	*Ocimum tenuiflorum*	−7.5	−7.5
4	IMPHY004479	Rhamnocitrin	C_16_H_12_O_6_	*Melissa officinalis*	−7.4	−7.4

### 3.4 ADME analysis

**FIGURE 3:**
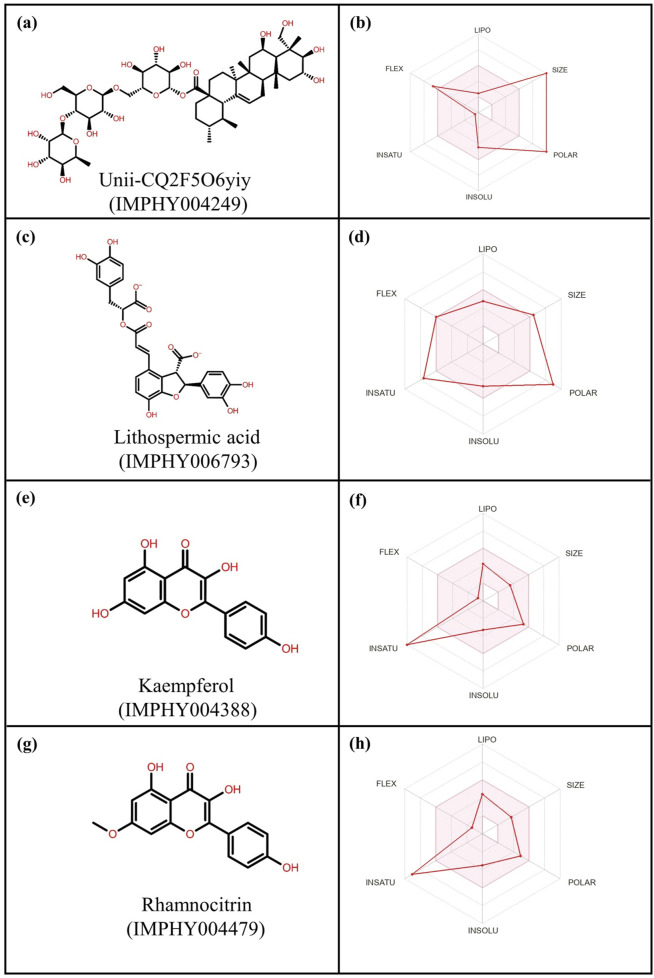
2-Dimensional structures and ADME profiling of the selected phytochemical compounds: **(a,b)** Unii-CQ2F5O6yiy; **(c,d)** lithospermic acid; **(e,f)** kaempferol; **(g,h)** rhamnocitrin.

This analysis helps in understanding intrinsic properties such as pharmacological properties, druglike nature, and medicinal chemistry friendliness (Anowar Hosen et al., 2022). These properties include absorption, distribution, metabolism, and excretion (Islam et al., 2022). Bioavailability is another important factor that contributes to making a compound a potential therapeutic, and various other parameters like blood–brain barrier (BBB), cytochrome inhibition activities, and permeability were used to check the therapeutic properties of compounds in the ADME analysis (Dwivedi et al., 2021). The top four compounds having the highest negative docking score were evaluated by uploading them on the SwissADME server, as shown in Figure 3 (Supplementary Table S3).

As per ADME analysis, all of the top four selected phytochemical compounds lack blood–brain barrier (BBB) permeability (Supplementary Table S3). These four compounds were found to be non-inhibitors of the CYP2C19 and CYP2C9 cytochromes, but two compounds, kaempferol and rhamnocitrin, were found to be inhibitors of the CYP1A2, CYP2D6, and CYP3A4 cytochromes. These two compounds did not violate Lipinski, Ghose, Veber, Egan, and Muegge rules to analyze drug-likeliness properties. Moreover, these two compounds exhibited an acceptable bioavailability score of 0.55, making them a favorable therapeutic drug. In contrast, Unii-CQ2F5O6yiy and lithospermic acid were found to be non-inhibitors of cytochromes CYP1A2, CYP2C19, CYP2C9, CYP2D6, and CYP3A4 and also showed low gastrointestinal absorption. Moreover, they violated drug-likeliness rules such as Lipinski, Ghose, Veber, Egan, and Muegge. However, these rules need not be strictly satisfied, because some drugs that violate these rules have been approved by the FDA (Pathania and Singh, 2021).

### 3.5 Redocking and intermolecular interaction analysis

**FIGURE 4:**
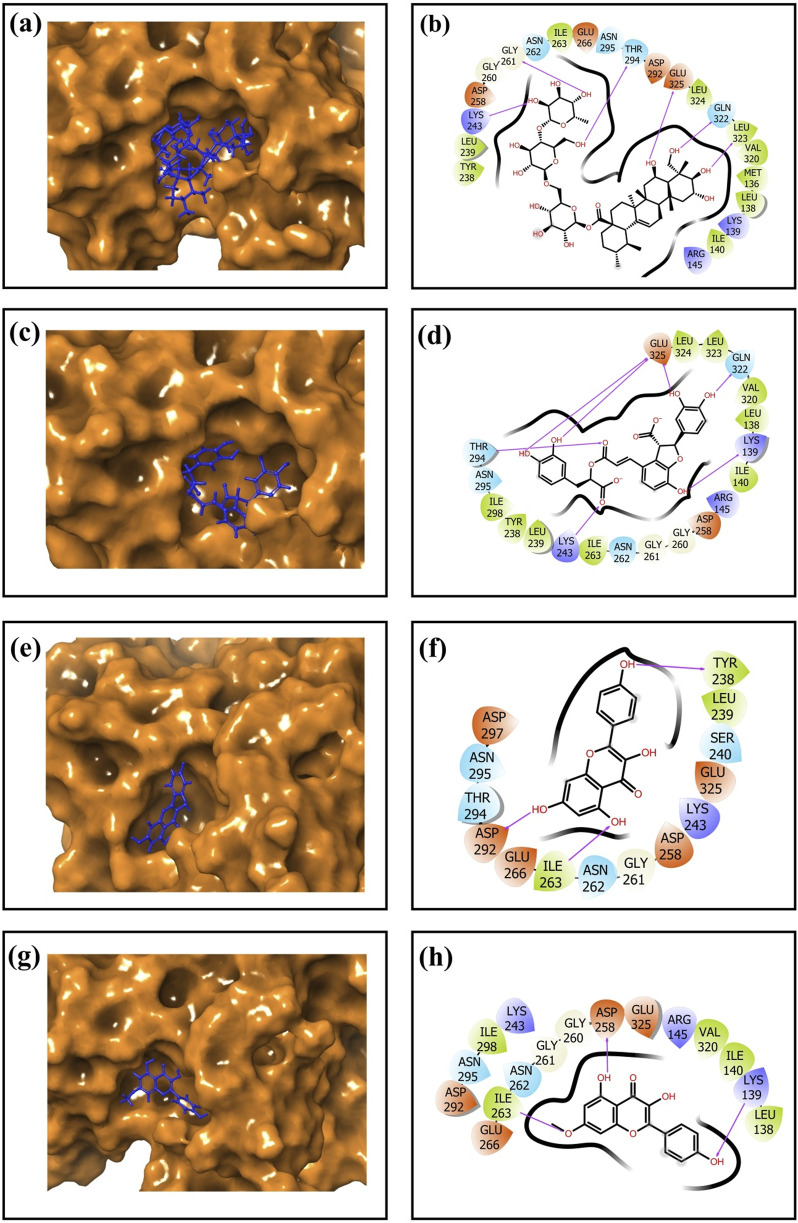
3-Dimensional and 2-dimensional docked complex poses of the selected phytochemical compounds: **(a,b)** Unii-CQ2F5O6yiy; **(c,d)** lithospermic acid; **(e,f)** kaempferol; **(g,h)** rhamnocitrin, showing binding on the active site of the cysteine proteinase. In the two-dimensional structures, H-bond formation (pink arrows), hydrophobic interaction (green), polar residue (blue), negative residual interaction (red), glycine (gray), and salt bridge (red and blue) interactions are logged for docked complexes of cysteine proteinase with the selected phytochemical compounds.

After virtual screening, the top ten phytochemical compounds were selected for further analysis of redocking based on their docking scores. Reference compound (TTP-6171) was also docked with cysteine proteinase in the same active site. The top ten compounds showed docking scores between −9.5 kcal/mol and −6.7 kcal/mol, whereas the reference compound showed a docking score of −9.1 kcal/mol. Unii-CQ2F5O6yiy revealed the most negative docking score of −9.5 kcal/mol. The molecular interactions were studied to evaluate the efficacy of the top four phytochemical compounds against the target cysteine proteinase and to understand their stability with respect to the target. The docked complex of cysteine proteinase-Unii-CQ2F5O6yiy showed a docking score of −9.5 kcal/mol, with six hydrogen bonds (Lys243, Gly261, Thr294, Gln322, Leu323, and Glu325 residues) (Table 2; Figures 4a,b), while cysteine proteinase-lithospermic acid had a docking score of −8.9 kcal/mol and formed five hydrogen bonds with Lys139, Lys243, Thr294, Gln322, and Glu325(2) residues (Table 2; Figures 4c,d). The docked complex of cysteine proteinase-kaempferol exhibited a docking score of −7.5 kcal/mol and formed three hydrogen bonds (Tyr238, Ile263, and Asp292 residues) (Table 2; Figures 4e,f). Cysteine proteinase-rhamnocitrin showed a docking score of −7.4 kcal/mol and formed three hydrogen bonds (Lys139, Asp258, and Ile263 residues) (Table 2; Figures 4g,h). The docking between cysteine proteinase and TTP-6171 (Control) showed a docking score of −9.1 kcal/mol and formed three hydrogen bonds (Arg145, Arg165, and Gly260 residues).

**TABLE 2:** Molecular interaction profiling of the top four selected phytochemical compounds: (a) Unii-CQ2F5O6yiy; (b) lithospermic acid; (c) kaempferol; (d) rhamnocitrin; (e) control (TTP-6171).

Sr no.	Complex name	H-bond	Hydrophobic	Polar	Positive	Negative	Salt bridge
1	Proteinase-Unii-CQ2F5O6yiy	Lys^243^, Gly^261^, Thr^294^, Gln^322^, Leu^323^, Glu^325^	**Met** ^**136**^, **Leu** ^**138**^ **, Ile140**, Tyr^238^, Leu^239^, **Ile** ^**263**^, **Val** ^**320**^, **Leu** ^**323**^, **Leu** ^**324**^	**Asn** ^**262**^ **, Thr** ^**294**^ **, Asn** ^**295**^ **, Gln** ^**322**^	**Lys** ^**139**^ **, Arg** ^**145**^ **,** Lys^243^	Asp^258^, Glu^266^, Asp^292^, **Glu** ^**325**^	-
2	Proteinase-lithospermic acid	Lys^139^, Lys^243^, Thr^294^, Gln^322^, Glu^325^,	**leu** ^**138**^, **Ile** ^**140**^, Tyr^238^, Leu^239^, **Ile** ^**263**^, Ile^298^, **Val** ^**320**^, **Leu** ^**323**^, **Leu** ^**324**^	**Asn** ^**262**^ **, Thr** ^**294**^ **, Asn** ^**295**^ **, Gln** ^**322**^	**Lys** ^**139**^ **, Arg** ^**145**^ **,** Lys^243^	Asp^258^, **Glu** ^**325**^	-
3	Proteinase-kaempferol	Tyr^238^, Ile^263^, Asp^292^	Tyr^238^, Leu^239^, **Ile** ^**263**^	Ser^240^, **Asn** ^**262**^ **, Thr** ^**294**^ **, Asn** ^**295**^	Lys^243^	Asp^258^, Glu^266^, Asp^292^, Asp^297^, **Glu** ^**325**^	-
4	Proteinase-rhamnocitrin	Lys^139^, Asp^258^, Ile^263^	**leu** ^**138**^, **Ile** ^**140**^, **Ile** ^**263**^, Ile^298^, **Val** ^**320**^	**Asn** ^**262**^ **, Asn** ^**295**^	**Lys** ^**139**^ **Arg** ^**145**^ **,** Lys^243^	Asp^258^, Glu^266^ Asp^292^, **Glu** ^**325**^	-
5	Proteinase-TTP-6171 (Control)	Arg^145^, Arg^165^, Gly^260^	Met^136^, Leu^138^, Ile^140^, Pro^163^, Trp^168^, Ile^263^, Val^320^, Leu^323^,Leu^324^	Asn^262^, Thr^294^, Asn^295^, Gln^322^	Lys^139^, Arg^145^, Lys^160^, Arg^165^	Asp^137^, Glu^162^, Glu^325^	Glu^162^, Arg^165^

The bold-highlighted residues are involved in interactions similar to those observed in the control complex.

These data suggested that the complexes cysteine proteinase-Unii-CQ2F5O6yiy and cysteine proteinase-lithospermic acid formed the maximum hydrogen bonds. The salt bridge was only observed in control TTP-6171 (Glu162 and Arg165 residues) and was not seen in any other docked complexes (Supplementary Figure S3a,S3b). Various other residual interactions were observed in the docked complex along with the reference ligand, which involve hydrophobic, positive, negative, and polar residues. Gly260 and Gly261 residues were present in the reference ligand and all the other docked complexes, except for cysteine proteinase-kaempferol, which showed a Gly261 residue only. The docked complexes also revealed the interactions with the same residues as those of the reference ligand, which probably signifies that they occupied the same binding pocket. These residues include Met136, Leu138, Ile140, Ile262, Val320, Leu323, and Leu324, which show hydrophobic interactions. All residues involved in the polar interaction (Asn262, Asn294, Asn295, and Gln322) were found in the docked complexes, including the reference molecule. The positive residues (Lys139 and Arg145) of the reference ligand were also found in the docked complexes. Negative residual interaction (Glu325) was present in all docked complexes. Overall, hydrogen bonding is often a dominant force in stabilizing protein-ligand complexes, so the docked complexes cysteine proteinase-Unii-CQ2F5O6yiy and cysteine proteinase-lithospermic acid were considered to be the most stable complexes, as they showed more H-bonding than the reference compound.

### 3.6 MD simulation analysis

MD simulation was performed at a 250 ns simulation time to predict the dynamic stability and intermolecular interactions of each docked cysteine proteinase with the phytochemical compounds, viz., Unii-CQ2F5O6yiy, lithospermic acid, kaempferol, rhamnocitrin, and analyzed compared to the reference docked complex, cysteine proteinase-TTP-6171.

**FIGURE 5:**
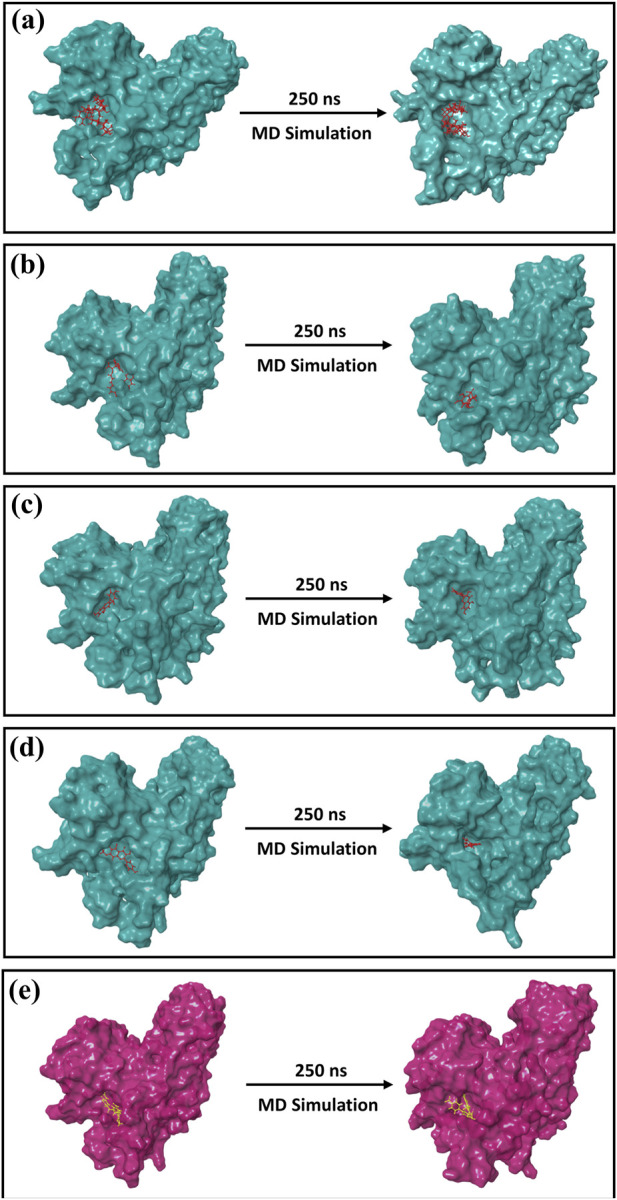
3D position of the first and last poses obtained from the 250-ns MD simulation for **(a)** Unii-CQ2F5O6yiy; **(b)** lithospermic acid; **(c)** kaempferol; **(d)** rhamnocitrin; **(e)** control TTP-6171.

The formation of the first and last docked poses of 3D structures is shown in Figure 5. By comparing the final poses of the 250-ns MD simulations with the corresponding docked poses, it was possible to ensure that the docked phytochemical compounds would remain as ligands in the binding pocket of the cysteine proteinase. A 3D surface analysis of the final poses from the 250-ns MD simulation shows acceptable conformational changes in the protein structure docked with phytochemical compounds against the cysteine proteinase docked with the TTP-6171 inhibitor, and also showed acceptable deviation in the conformation of the selected phytochemical compounds, indicating that the docked phytochemical compounds could be potential inhibitors of the cysteine proteinase compared to the reference compound (Figure 5).

#### 3.6.1 Root mean square deviation (RMSD) analysis

**FIGURE 6:**
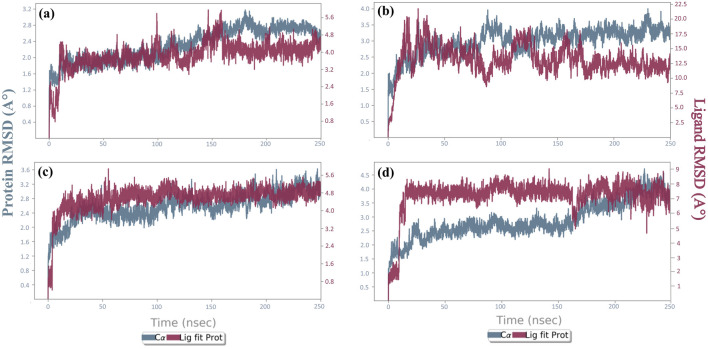
RMSD plots for the backbone atoms of cysteine proteinase and selected phytochemical compounds: **(a)** Unii-CQ2F5O6yiy; **(b)** lithospermic acid; **(c)** kaempferol; **(d)** rhamnocitrin. The fits on the selected target protein were extracted from 250-ns MD simulation trajectories of different docked complexes.

The deviation of the system from the first frame was measured by RMSD over the trajectory. To determine the protein-ligand complexes equilibration stage, RMSD was computed throughout the entire trajectory for the protein and the ligand, independently. A conformational variance (<3 Å) was regarded as acceptable. The lines in the RMSD graph were parallel to the X-axis and show minimum deviation. The RMSDs of every screened compound, as well as the control compound, over the 250-ns trajectory are displayed in Figure 6 (Supplementary Figure S4a). The protein RMSD for the complexes cysteine proteinase-Unii-CQ2F5O6yiy and cysteine proteinase-kaempferol was <3.5 Å, and cysteine proteinase-lithospermic acid and cysteine proteinase-rhamnocitrin complexes showed RMSDs of 4 Å and 4.5 Å, respectively. The reference complex showed an RMSD of 4.5 Å. The RMSD of Unii-CQ2F5O6yiy in the protein–ligand complex exhibited slight deviation until 10 ns of the initial stage of MD simulation, where the RMSD reached 2 Å, and then it became stable until 100 ns. After 100 ns, it showed a slight deviation but remained stable throughout the remaining simulation time. The protein in the cysteine proteinase-Unii-CQ2F5O6yiy complex showed stability with RMSD (2.1 Å) until 125 ns. After that, it reached 3 Å during the remaining simulation time, as shown in Figure 6a. However, the phytochemical compound lithospermic acid in the respective complex showed a substantial deviation (10 Å to 22 Å), which is not acceptable. In contrast, the protein in the complex exhibited a stable RMSD (3.8 Å) during the total simulation time (Figure 6b). Here, the protein and ligand in the cysteine proteinase-kaempferol complex showed stable and consistent RMSDs of 3.5 Å and 5.4 Å after 5 ns of the simulation time, respectively (Figure 6c). In the cysteine proteinase-rhamnocitrin complex, the phytochemical compound rhamnocitrin showed steep rise from 2 Å to 8 Å after 10 ns of total simulation time but exhibited stability and consistency in RMSD after 10 ns, whereas protein in the respective complex had a stable and acceptable RMSD of 3 Å until 170 ns, and then it exhibited a slight rise in its RMSD and reached 4.5 Å during the remaining 80 ns of the molecular docking simulation (MDS) (Figure 6d). The reference inhibitor TTP-6171 in the cysteine proteinase-TTP-6171 complex showed an RMSD of 4 Å until 40 ns, and then a gradual increase in RMSD from 4 Å to 6.6 Å of the inhibitor was noted after 40 ns to the end of the simulation time. Similarly, the protein in the respective complex displayed a slight fluctuation until 40 ns, then showed a stable RMSD after 40 ns at 4 Å, as shown in Supplementary Figure S4a. Based on the RMSDs of the selected phytochemicals, all the candidates displayed acceptable stability when docked with the receptor protein cysteine proteinase, except for lithospermic acid. This was further supported by the RMSF calculations of the protein and selected compounds.

#### 3.6.2 Root mean square fluctuation (RMSF) analysis

The RMSF calculation is crucial for the analysis of local fluctuations between protein chains and selected docked compounds. The local fluctuations in the target protein were produced by the amino acid residues, and fluctuations produced in the phytochemical compounds were due to atoms of the selected compounds that were involved in the docking process. Interestingly, the amino acid residues of the target protein showed acceptable fluctuations of <4.8 Å and showed one peak (residues at the 150 position) in all the complexes. In the cysteine proteinase-lithospermic acid and cysteine proteinase-rhamnocitrin complexes, the residues of the target protein gave >6.4 Å RMSF at the peak 150 position. The amino acid residues in the reference complex showed fluctuations of 5.6 Å (Supplementary Figure S5). The RMSF calculated for the selected phytochemical compounds fit in the active site of the cysteine proteinase with respect to the total MD simulation time, suggesting an acceptable deviation of <4 Å by the atoms of all the selected phytochemicals except for lithospermic acid, which exhibited an RMSF <8 Å (Supplementary Figure S6). In the reference complex, cysteine proteinase-TTP-6171, the amino acid residues of cysteine proteinase exhibited an insignificant deviation with RMSF >5.6 Å, whereas cysteine proteinase in all the docked complexes displayed less fluctuations except in the complex with lithospermic acid. Additionally, atoms of TTP-6171 in the reference complex showed acceptable fluctuations <4 Å, whereas atoms of Unii-CQ2F5O6yiy and kaempferol in the docked complexes exhibited less fluctuations than the reference compound, which confirms the stability of the compounds (Supplementary Figures S4b,S4c,S5,S6). These RMSF and RMSD data provided support for the integration of potential compounds within the binding site of the cysteine proteinase target.

#### 3.6.3 Protein–ligand interaction mapping

The non-covalent interactions, particularly H-bonds and other interactions including hydrophobic and ionic contacts, π–π stacking, salt bridges, and water bridge formation, were identified as crucial forces to maintain the stability of the complex in the protein–ligand interaction. Protein–ligand contact profiling based on non-covalent interactions was measured to compare all the cysteine proteinase phytochemical compound complexes to the reference complex.

**FIGURE 7:**
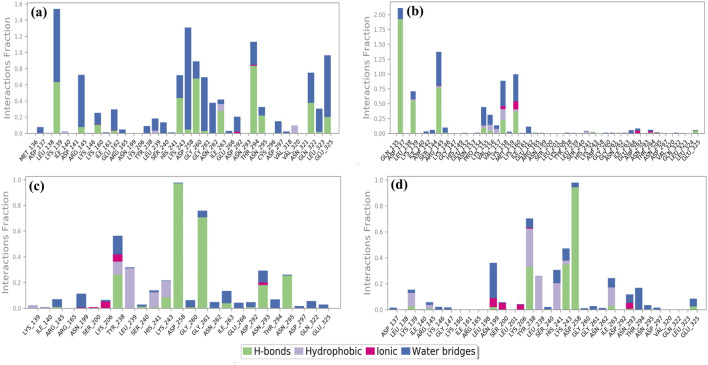
Protein–ligand interaction mapping for cysteine proteinase docked with selected phytochemical compounds: **(a)** Unii-CQ2F5O6yiy; **(b)** lithospermic acid; **(c)** kaempferol; **(d)** rhamnocitrin, extracted from 250-ns molecular dynamics simulations.

Cysteine proteinase-Unii-CQ2F5O6yiy showed hydrogen bond formation with residues Lys^139^, Lys^243^, Gly^260^, Thr^294,^ and Gln^322^ for more than 50% of total simulation time, whereas residues Lys^139^, Arg^145^, Asp^258^, Gly^261,^ and Glu^325^ exhibited formation of water bridges for more than 60% of the simulation, and Ile^263^ and Val^320^ noted hydrophobic interaction for less than 25% of total MD simulation time. Some other residues also showed hydrogen bonding and formation of water bridges for a relatively shorter period of simulation time (Figure 7a). Likewise, the cysteine proteinase–lithospermic acid docked complex formed a hydrogen bond with Asp^137^ for more than 100% of the simulation time, and residues Lys^139^, Arg^145^, and Lys^160^ were observed for more than 40% of the total simulation interval. Arg^145^, Lys^160^, and Lys^160^ formed water bridges for 40% of the simulation period. Additionally, some other residues were detected for other intermolecular interactions (Figure 7b). The cysteine proteinase–kaempferol complex exhibited hydrogen bonding with Asp^258^ and Gly^261^ residues for more than 70% of the simulation time, while Tyr^238^ and Lys^243^ residues were observed for more than 20% of the total interval of the MD simulation. Tyr^238^, Leu^239^, His^241^, and Lys^243^ showed hydrophobic interaction with the docked ligand for more than 15% of the simulation interval. Multiple residues formed water bridges for less than 20% of the simulation, along with the hydrogen bond, and some residues showed ionic interaction for a very short interval (Figure 7c). In the analysis of protein–ligand contact mapping of the cysteine proteinase–rhamnocitrin docked complex, Asp^258^ exhibited hydrogen bond formation at 100% of the simulation interval, whereas Tyr^238^, Leu^239^, and His^241^ residues showed more than 20% hydrophobic interaction during the total simulation period. Notably, the Asn^199^ residue showed more than 30% of the water bridge interaction along with the ionic bond (Figure 7d). In the protein–ligand interaction mapping of the reference complex, cysteine proteinase-TTP-6171, Lys^139^ and Asn^29^ residues were noted for hydrogen bond formation for more than 50% of the simulation time, whereas His^241^ exhibited hydrophobic interaction for more than 100% of the MD simulation time. Water bridges were formed with residues Gly^261^ for 100% and Asp^258^ and Glu^325^ for more than 40% of the simulation time (Supplementary Figure S7a). In the context of these analyses of complexes, selected phytochemical compounds can be utilized as effective inhibitors of the target cysteine proteinase, due to their high stability at the active pocket of cysteine proteinase involving the formation of strong hydrogen bonds, water bridges, and hydrophobic interactions.

**FIGURE 8:**
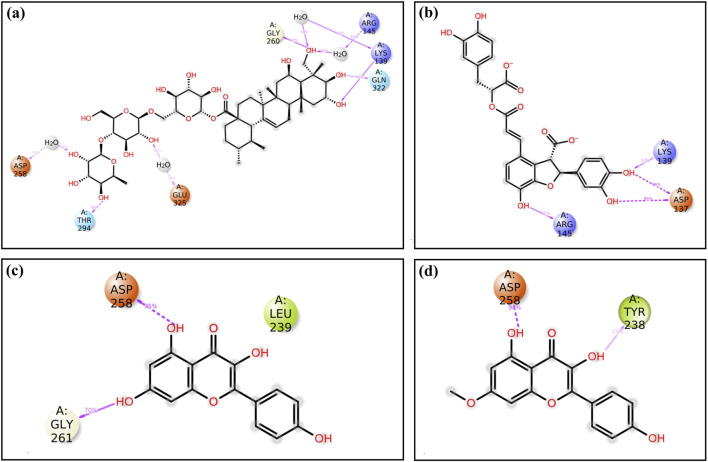
A detailed schematic representation of the atomic interaction of the ligands of the selected phytochemical compounds: **(a)** Unii-CQ2F5O6yiy; **(b)** lithospermic acid; **(c)** kaempferol; **(d)** rhamnocitrin, docked with cysteine proteinase. Interactions that occur for more than 30.0% of the simulation period (0.00 ns through 250.04 ns) are displayed on the chosen trajectory.

Additionally, during a 250-ns simulation, the intermolecular interactions between the residues of cysteine proteinase and Unii-CQ2F5O6yiy, lithospermic acid, kaempferol, rhamnocitrin, and TTP-6171 were calculated at the 30% interval of the 250-ns MD simulation. This revealed a significant binding of the corresponding ligands with active residues (Figure 8; Supplementary Figure S7b). The stability of the chosen compounds at the active site of cysteine proteinase was suggested by the observation of hydrogen bonding and water bridge interactions for all of the chosen ligands. Therefore, docked complexes can be organized in order of stability using the 250-ns molecular dynamics simulation analysis: cysteine proteinase-Unii-CQ2F5O6yiy, cysteine proteinase-kaempferol, cysteine proteinase-lithospermic acid, and cysteine proteinase-rhamnocitrin.

### 3.7 Binding free energy analysis

**FIGURE 9:**
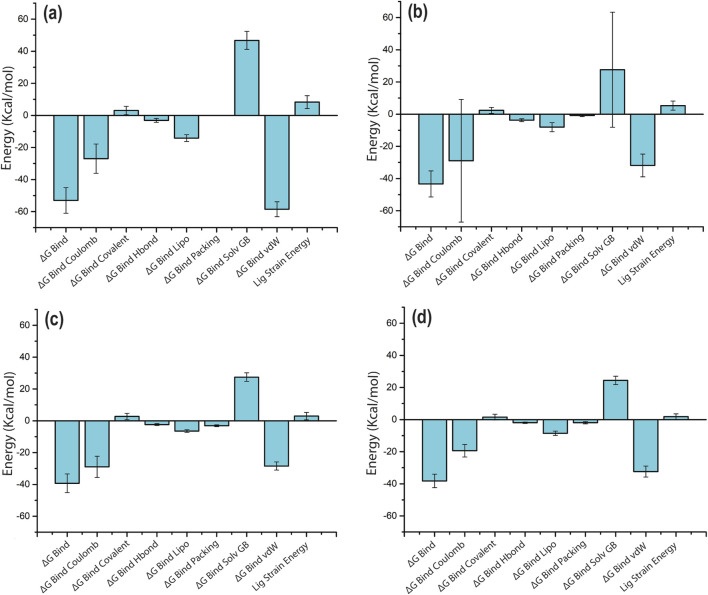
MM/GBSA analysis of per-frame binding free energy and energy components values for selected phytochemical compounds complexed with cysteine proteinase: **(a)** Unii-CQ2F5O6yiy; **(b)** lithospermic acid; **(c)** kaempferol; **(d)** rhamnocitrin.

The docking score of the top four selected phytochemical compounds, in contrast with the reference ligand, was further investigated using the binding free energy analysis. The net binding free energy and each energy component per frame that contributes to the stability of potential phytochemicals compared to TTP-6171 (reference molecule) located in the binding pocket of cysteine proteinase were calculated using the MM/GBSA module of the Schrödinger suite. The MM/GBSA binding free energies per frame (mean ± standard deviation) of the four compounds and the reference compound are displayed in Supplementary Table S4. All four compounds showed a significant binding free energy, demonstrating that the chosen compounds had a good docking score for the receptor. It is noteworthy that the ΔGBind for the reference complex was −44.06 ± 4.97 kcal/mol, whereas the ΔGBind for the four selected compounds ranged from −53.02 ± 7.9 kcal/mol to −38.22 ± 4.2 kcal/mol (Figure 9; Supplementary Figure S8; Supplementary Table S4). Additionally, the cysteine proteinase–Unii-CQ2F5O6yiy complex showed significantly higher MM/GBSA binding free energy than the reference complex. The stability of each complex of the cysteine proteinase phytochemical compound was significantly influenced by the ΔGBind Coulomb and ΔGBind vdW. These results indicated that Unii-CQ2F5O6yiy, lithospermic acid, kaempferol, and rhamnocitrin have a high affinity for the cysteine proteinase protein. Therefore, these binding free energy values per frame supported the hypothesis that phytochemical compounds identified through the screening procedure could function as cysteine proteinase inhibitors for the management of monkeypox.

An earlier study focused on screening microbially derived natural compounds and repurposing tetracyclines to identify potential inhibitors targeting the cysteine proteinase of monkeypox virus (MPXV) at its binding site, while our study expanded its scope to include phytochemicals derived from specific plants. Phytochemicals sourced from specific plants tend to be more accessible and cost-effective than microbially derived compounds or specialized antibiotics like tetracyclines. This accessibility could make the identified inhibitors more practical for further development and potential therapeutic applications (Singh et al., 2023).

Several microbially derived natural compounds, such as gallicynoic acid F (NPA002071), H2-erythro-neopterin (NPA000530), nigcollin C (NPA029767), NPA24545, and vaccinol M (NPA030378), have exhibited good docking against the cysteine proteinase of MPXV with binding free energies ranging from −60.87 to −44.42 kcal/mol (Dubey et al., 2023). Additionally, strong docking has been shown for various stereoisomers of tetracycline with cysteine proteinase and DNA-dependent RNA polymerase (DdRp). In this study, the authors virtually screened 16 stereoisomers of tetracycline and identified tigecycline and eravacycline as the top stereoisomer candidates that exhibit the highest docking score against MPXV DNA-dependent RNA polymerase with docking scores of −8.88 kcal/mol and −7.87 kcal/mol, respectively. Additionally, the stereoisomers omadacycline and minocycline showed notable docking scores of −10.6 kcal/mol and −7.51 kcal/mol against cysteine proteinase (Alandijany et al., 2023). Compounds isocinchophyllamine (Ghate et al., 2024), SC75741, ammonium glycyrrhizinate (Rabaan et al., 2024), CHEMBL32926, and CHEMBL4861364 (Imran et al., 2023) were also reported against the cysteine proteinase of MPXV. Whereas in the current study, the phytochemical compound Unii-CQ2F5O6yiy exhibited a stronger docking score than minocycline and a slightly smaller docking score than omadacycline (Alandijany et al., 2023). The A42R profilin-like protein of MPXV involved in cell development and motility was used as a drug target in a study by Ashley et al. (2024). They used a library of 36,366 compounds from Traditional Chinese Medicine TCM, AfroDb, and PubChem databases for virtual screening against A42R profilin-like protein and identified seven compounds (PubChem CID: 11371962, ZINC000000899909, ZINC000001632866, ZINC000015151344, ZINC000013378519, ZINC000000086470, and ZINC000095486204) with favorable binding affinities (−7.2 to −8.3 kcal/mol) compared to tecovirimat (−6.7 kcal/mol) (Ashley et al., 2024). In contrast, our top four compounds showed docking scores of −9.5 to −7.4 kcal/mol against MPXV cysteine proteinase protein. Here, Unii-CQ2F5O6yiy, lithospermic acid, kaempferol, and rhamnocitrin have shown promising docking characteristics compared to the reference compound TTP-6171 against MPXV cysteine proteinase. Remarkably, among these compounds, Unii-CQ2F5O6yiy demonstrated exceptional promise as it exhibited a docking score of −9.5 kcal/mol against MPXV cysteine proteinase protein. Hence, this compound stands out as a prime candidate for further validation studies, suggesting its potential as an effective inhibitor of cysteine proteinase in combating MPXV infections.

## 4 Conclusion

MPXV cysteine proteinase plays a key role in the viral replication process. Therefore, inhibiting cysteine proteinase could be important for the management of MPXV. In view of this, we identified four phytochemical compounds, Unii-CQ2F5O6yiy, lithospermic acid, kaempferol, and rhamnocitrin, through *in silico* study. The compounds were found to have docking scores ranging from −9.5 kcal/mol to −7.4 kcal/mol. The RMSD of proteins in all the phytochemical complexes exhibited acceptable deviation except for lithospermic acid, whereas the atoms of Unii-CQ2F5O6yiy and kaempferol in the docked complexes exhibited less fluctuation than the reference compound (<5.4 Å). Notably, Unii-CQ2F5O6yiy demonstrated the highest inhibiting potential among these compounds against MPXV cysteine proteinase. Hence, compound Unii-CQ2F5O6yiy may be utilized as a potential drug in the future for the management of MPXV. Findings from this study are encouraging, although further experimental validation of this compound is required under *in vitro* and *in vivo* conditions against MPXV to assess its drug potential.

## Data Availability

The original contributions presented in the study are included in the article/Supplementary Material; further inquiries can be directed to the corresponding author.
