# Supplementary material for: Computational investigation of potential natural compounds as inhibitors of monkeypox virus cysteine proteinase
Source: Front Bioinform. 2025 Jul 28;5:1637207. doi: 10.3389/fbinf.2025.1637207 (PMC12336155; doi:10.3389/fbinf.2025.1637207)
Supplement: Supplementary file 1 [file DataSheet1.docx]

**Results and Discussion**

**S1.1 Structure based virtual screening**

**Table S1.** Final phytochemical compounds for virtual screening obtained after deletion of duplicate compounds downloaded from IMPPAT database.

| **Plants name** | **Compounds obtained from IMPPAT** | **Duplicate Compounds** | **Compounds obtained after deletion of Duplication** |
| --- | --- | --- | --- |
| *Melissa officinalis* (Lemon balm) | 473 | 249 | 224 |
| *Ocimum tenuiflorum* (Tulsi) | 593 | 341 | 252 |
| *Mentha arvensis* (Wild mint) | 482 | 298 | 184 |
| *Mentha piperita* (Peppermint) | 649 | 358 | 291 |
| *Pogostemon cablin* (Patchouli) | 334 | 195 | 139 |
| **Total** | 2531 | 1441 | 1090 |
| **Final compounds for VS** | **1090** | **521** | **569** |

**Table S2.** List of virtual screened phytochemical compounds against Cysteine proteinase of *Monkeypox virus*.

| **S.NO** | **IMPPAT PHYTOCHEMICAL ID** | **PHYTOCHEMICAL NAME** | **Energy** |
| --- | --- | --- | --- |
| 1 | IMPHY004249 | Unii-CQ2F5O6yiy | -9.3 |
| 2 | IMPHY006793 | Lithospermic acid | -8.8 |
| 3 | IMPHY004388 | Kaempferol | -7.5 |
| 4 | IMPHY004479 | Rhamnocitrin | -7.4 |
| 5 | IMPHY003487 | 3-methylalizarin | -7.3 |
| 6 | IMPHY003883 | Labd-14-ene, 8,13-epoxy-, (13S)- | -7.1 |
| 7 | IMPHY004752 | 2-Methoxyanthraquinone | -6.9 |
| 8 | IMPHY005380 | Hymenoxin | -6.9 |
| 9 | IMPHY006188 | Caftaric acid | -6.8 |
| 10 | IMPHY003987 | Eugenyl glucoside | -6.7 |
| 11 | IMPHY003392 | Butylated hydroxytoluene | -6.4 |
| 12 | IMPHY006371 | pogopyrone b | -6.4 |
| 13 | IMPHY004352 | Patchoulenone | -6.3 |
| 14 | IMPHY000061 | Patchouli alcohol | -6.3 |
| 15 | IMPHY005940 | Thunbergol | -6.2 |
| 16 | IMPHY001905 | Bisabolol oxide B | -6.1 |
| 17 | IMPHY004281 | Guaiol | -6.1 |
| 18 | IMPHY003695 | (-)-Germacrene A | -6 |
| 19 | IMPHY003398 | Myristicin | -6 |
| 20 | IMPHY000005 | Thiamine | -5.9 |
| 21 | IMPHY004120 | (-)-Cyperene | -5.9 |
| 22 | IMPHY005618 | Germacrene B | -5.9 |
| 23 | IMPHY004286 | Longifolene | -5.9 |
| 24 | IMPHY002558 | Mintsulfide | -5.8 |
| 25 | IMPHY004971 | alpha-Bourbonene | -5.8 |
| 26 | IMPHY000573 | Chamazulene | -5.7 |
| 27 | IMPHY000112 | Isophytol | -5.4 |
| 28 | IMPHY001931 | Vanillin | -5.4 |
| 29 | IMPHY003798 | (Z)-alpha-Bisabolene | -5.4 |
| 30 | IMPHY003490 | Coumarin | -5.4 |
| 31 | IMPHY006534 | (Z)2,(E)4,(E)6-Allofarnesene | -5.4 |
| 32 | IMPHY003495 | 2-Methoxy-4-vinylphenol | -5.4 |
| 33 | IMPHY002913 | Sesquisabinene | -5.3 |
| 34 | IMPHY006486 | Squalene | -5.3 |
| 35 | IMPHY003536 | Eugenol | -5.3 |
| 36 | IMPHY004536 | Geranic acid | -5.2 |
| 37 | IMPHY004135 | alpha-elemene | -5.2 |
| 38 | IMPHY003296 | Piperitenone | -5.2 |
| 39 | IMPHY004195 | Neomenthyl acetate | -5 |
| 40 | IMPHY004077 | Verbenone | -5 |
| 41 | IMPHY006419 | 6-Methyl-3,5-heptadien-2-one | -5 |
| 42 | IMPHY006944 | Estragole | -4.9 |
| 43 | IMPHY000915 | Chrysanthenone | -4.9 |
| 44 | IMPHY005489 | 2-Methylhexanoic acid | -4.9 |
| 45 | IMPHY006177 | Methyl geranate | -4.9 |
| 46 | IMPHY004528 | 1,3-p-Menthadien-7-al | -4.8 |
| 47 | IMPHY006948 | beta-Terpineol | -4.8 |
| 48 | IMPHY004225 | Methyl linoleate | -4.8 |
| 49 | IMPHY003545 | 4-Isopropylbenzaldehyde | -4.7 |
| 50 | IMPHY006352 | Nerol oxide | -4.7 |
| 51 | IMPHY004003 | Neoisopulegol | -4.7 |
| 52 | IMPHY006145 | p-Cymene | -4.7 |
| 53 | IMPHY006959 | Hydroquinone | -4.7 |
| 54 | IMPHY001658 | Thymol methyl ether | -4.6 |
| 55 | IMPHY000491 | Pinene | -4.6 |
| 56 | IMPHY006950 | Tricyclene | -4.6 |
| 57 | IMPHY004151 | Geranyl formate | -4.6 |
| 58 | IMPHY003238 | Isopulegone | -4.6 |
| 59 | IMPHY003525 | Nonanal | -4.6 |
| 60 | IMPHY006970 | Decanal | -4.5 |
| 61 | IMPHY003533 | 2-Methylanisole | -4.5 |
| 62 | IMPHY006070 | Styrene | -4.5 |
| 63 | IMPHY000545 | O-Cymene | -4.4 |
| 64 | IMPHY004541 | 3-Octyl acetate | -4.4 |
| 65 | IMPHY006972 | 1-Octacosanol | -4.4 |
| 66 | IMPHY006428 | (R)-Lavandulol | -4.4 |
| 67 | IMPHY006951 | Eicosane | -4.3 |
| 68 | IMPHY005345 | 1-Octen-3-OL | -4.3 |
| 69 | IMPHY005127 | Hexyl butyrate | -4.2 |
| 70 | IMPHY000795 | Octanal | -4.2 |
| 71 | IMPHY005390 | 1-Octen-3-yl acetate | -4.2 |
| 72 | IMPHY006337 | 1-Octen-3-one | -4.1 |
| 73 | IMPHY001896 | Heptacosane | -4.1 |
| 74 | IMPHY000060 | Myristic acid | -4 |
| 75 | IMPHY003485 | Myrcene | -3.9 |

**S1.2. Molecular dynamics simulation analysis of apo protein**


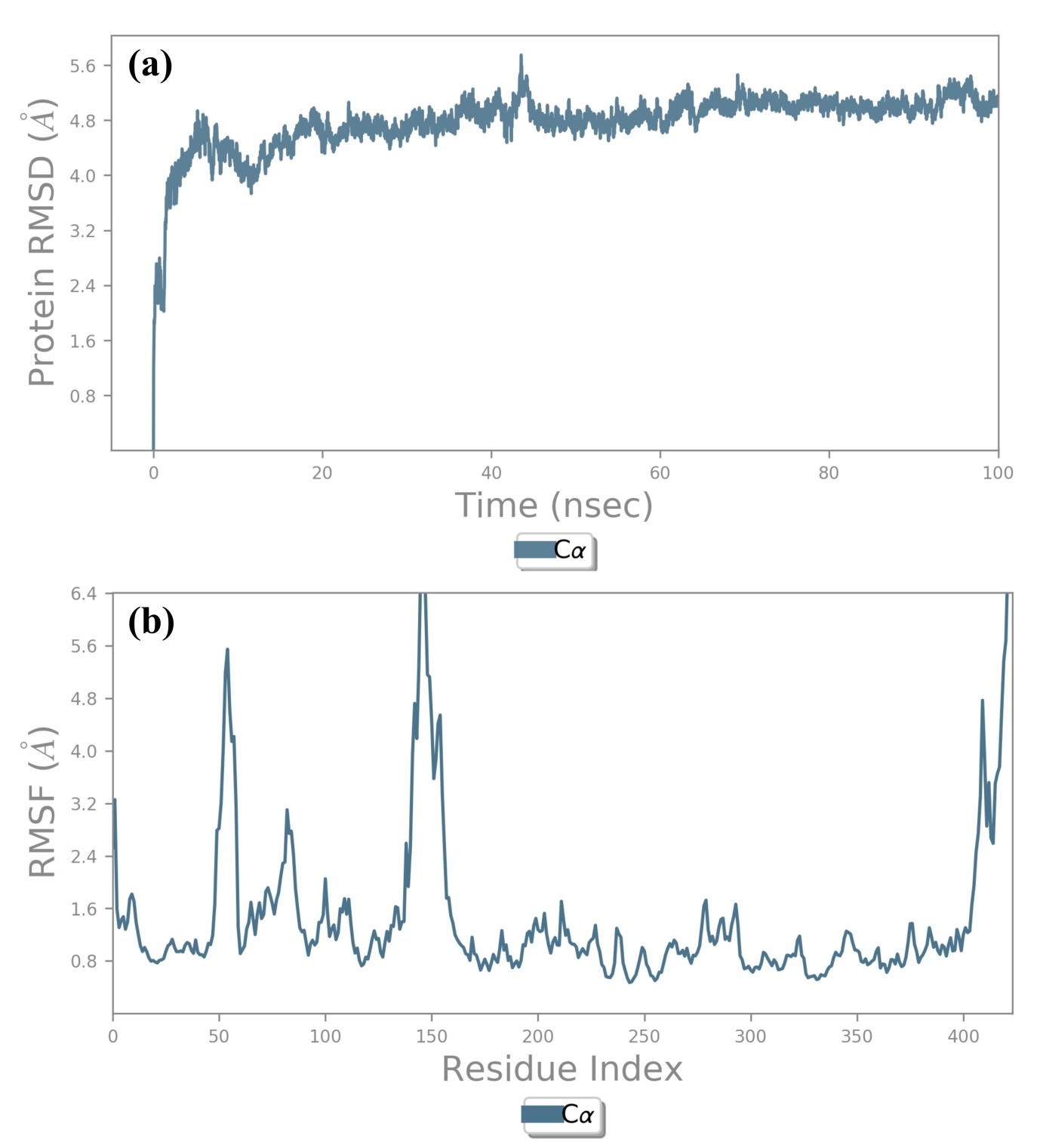


**Figure S1.** (a) RMSD plot for the backbone atoms of apo protein, (b) RMSF plot generated for the apo protein during 100 ns molecular dynamics simulation interval.

**S1.3. ADME profiling**

**Table S3.** ADME analysis for the top four selected phytochemical compounds against Cysteine proteinase.

| **Properties** | **Unii-CQ2F5O6yiy** | **Lithospermic acid** | **Kaempferol** | **Rhamnocitrin** |
| --- | --- | --- | --- | --- |
| iLOGP | 1.87 | 0.67 | 1.7 | 2.31 |
| XLOGP3 | -1.24 | 2.78 | 1.9 | 2.22 |
| WLOGP | -2.06 | 2.34 | 2.28 | 2.59 |
| MLOGP | -2.84 | 0.45 | -0.03 | 0.22 |
| Silicos-IT Log P | -2.53 | 1.85 | 2.03 | 2.55 |
| Consensus Log P | -1.36 | 1.62 | 1.58 | 1.98 |
| ESOL Log S | -4.44 | -4.68 | -3.31 | -3.51 |
| ESOL Solubility (mg/ml) | 3.50E-02 | 1.13E-02 | 1.40E-01 | 9.36E-02 |
| ESOL Solubility (mol/l) | 3.59E-05 | 2.10E-05 | 4.90E-04 | 3.12E-04 |
| ESOL Class | Moderately soluble | Moderately soluble | Soluble | Soluble |
| Ali Log S | -5.31 | -6.87 | -3.86 | -3.96 |
| Ali Solubility (mg/ml) | 4.79E-03 | 7.22E-05 | 3.98E-02 | 3.31E-02 |
| Ali Solubility (mol/l) | 4.91E-06 | 1.34E-07 | 1.39E-04 | 1.10E-04 |
| Ali Class | Moderately soluble | Poorly soluble | Soluble | Soluble |
| Silicos-IT LogSw | 1.52 | -3.14 | -3.82 | -4.52 |
| Silicos-IT Solubility (mg/ml) | 3.20E+04 | 3.87E-01 | 4.29E-02 | 9.07E-03 |
| Silicos-IT Solubility (mol/l) | 3.28E+01 | 7.18E-04 | 1.50E-04 | 3.02E-05 |
| Silicos-IT class | Soluble | Soluble | Soluble | Moderately soluble |
| GI absorption | Low | Low | High | High |
| BBB permeant | No | No | No | No |
| Pgp substrate | Yes | No | No | No |
| CYP1A2 inhibitor | No | No | Yes | Yes |
| CYP2C19 inhibitor | No | No | No | No |
| CYP2C9 inhibitor | No | No | No | No |
| CYP2D6 inhibitor | No | No | Yes | Yes |
| CYP3A4 inhibitor | No | No | Yes | Yes |
| log Kp (cm/s) | -13.13 | -7.61 | -6.7 | -6.56 |
| Lipinski #violations | 3 | 3 | 0 | 0 |
| Ghose #violations | 4 | 2 | 0 | 0 |
| Veber #violations | 1 | 1 | 0 | 0 |
| Egan #violations | 1 | 1 | 0 | 0 |
| Muegge #violations | 5 | 3 | 0 | 0 |
| Bioavailability Score | 0.17 | 0.11 | 0.55 | 0.55 |
| PAINS #alerts | 0 | 1 | 0 | 0 |
| Brenk #alerts | 1 | 2 | 0 | 0 |
| Leadlikeness #violations | 2 | 2 | 0 | 0 |
| Synthetic Accessibility | 10 | 4.98 | 3.14 | 3.21 |

**S1.4. Re-docking and intermolecular interaction analysis**

**
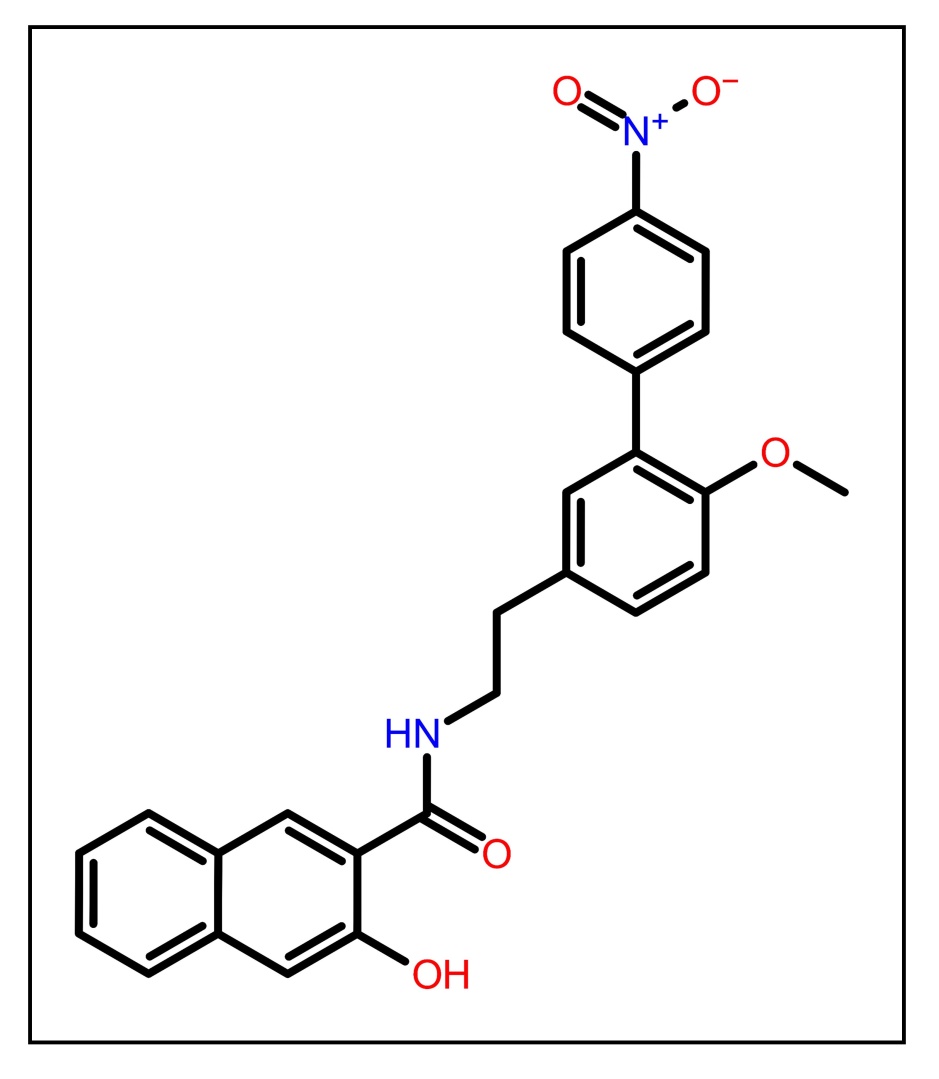
**

**Figure S2.** 2D structure of reference compound TTP-6171.


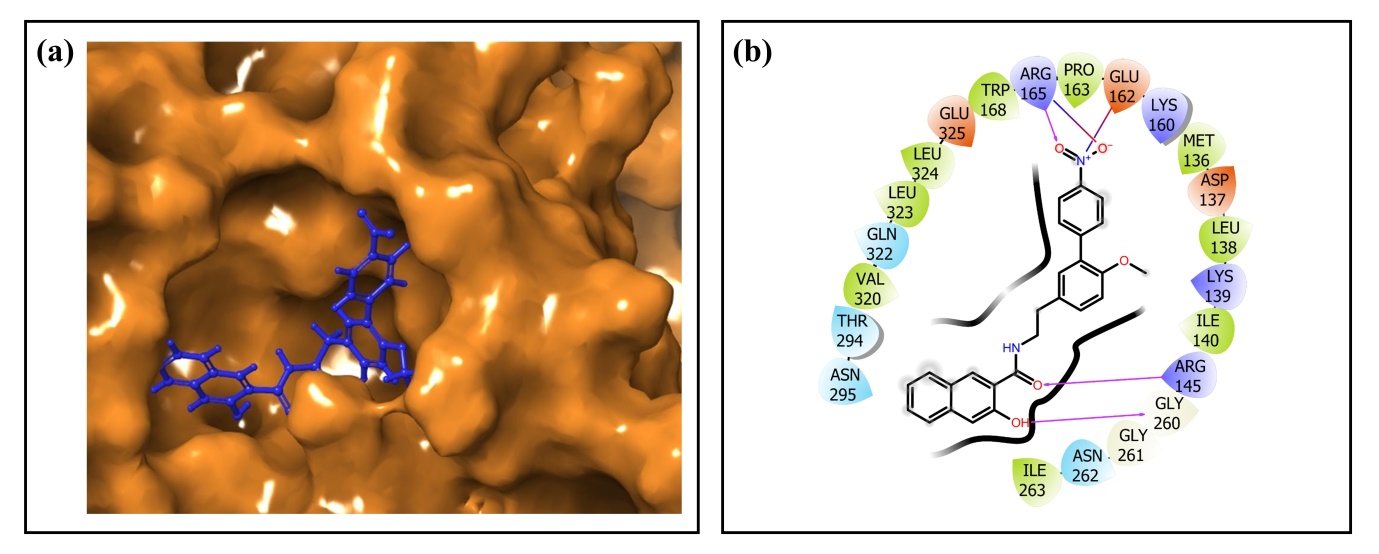


**Figure S3.** 3D and 2D docked complex poses of the reference complex, i.e., proteinase-TTP-6171 complex. In 2D structures, H-bond formation (pink arrows), hydrophobic interaction (green), polar residue (blue), negative residual interaction (red), glycine (grey) and salt bridge (red and blue) interactions are logged for docked complex.

**S1.5. Molecular dynamics simulation analysis**


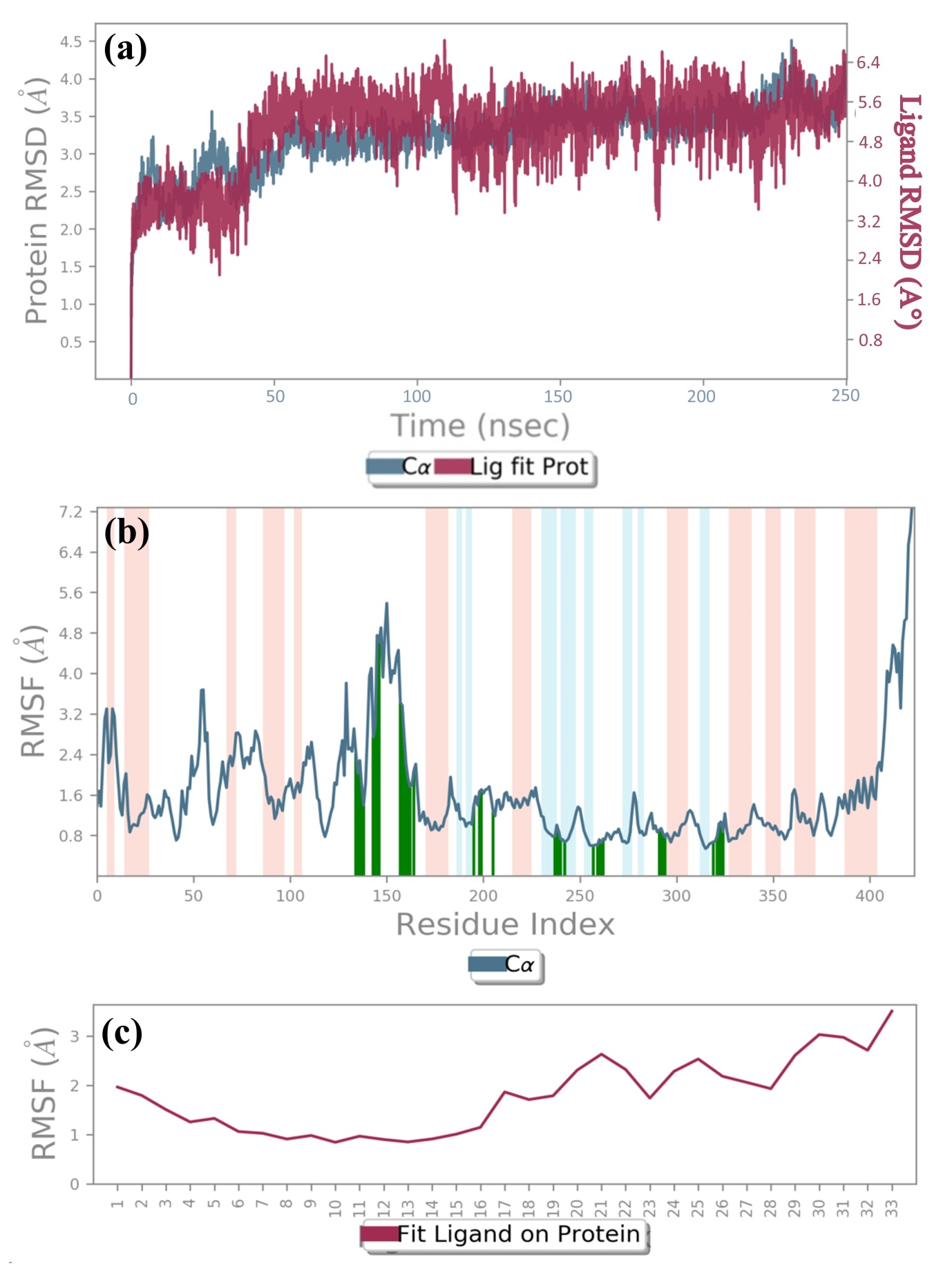


**Figure S4.** (a) RMSD plot for the backbone atoms of cysteine proteinase in complex with the reference molecule, i.e., TTP-6171, (b) RMSF plot generated for cysteine proteinase docked with reference ligand during 250 ns molecular dynamics simulation interval, (c) RMSF plot generated for the docked reference compound fit in the cysteine proteinase during 250ns molecular dynamics simulation interval.


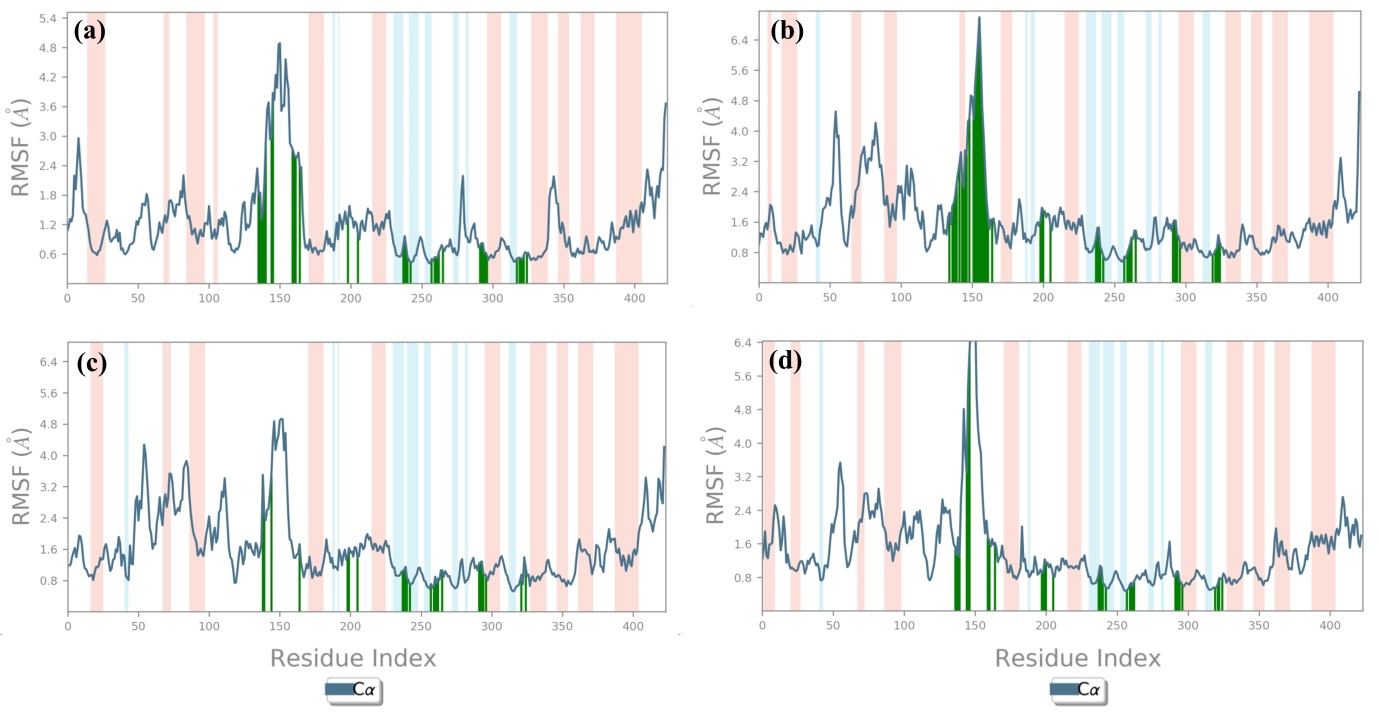


**Figure S5.** RMSF Plot generated for the cysteine proteinase docked with selected phytochemical compounds, i.e., (a) Unii-CQ2F5O6yiy, (b) Lithospermic acid, (c) Kaempferol and (d) Rhamnocitrin.


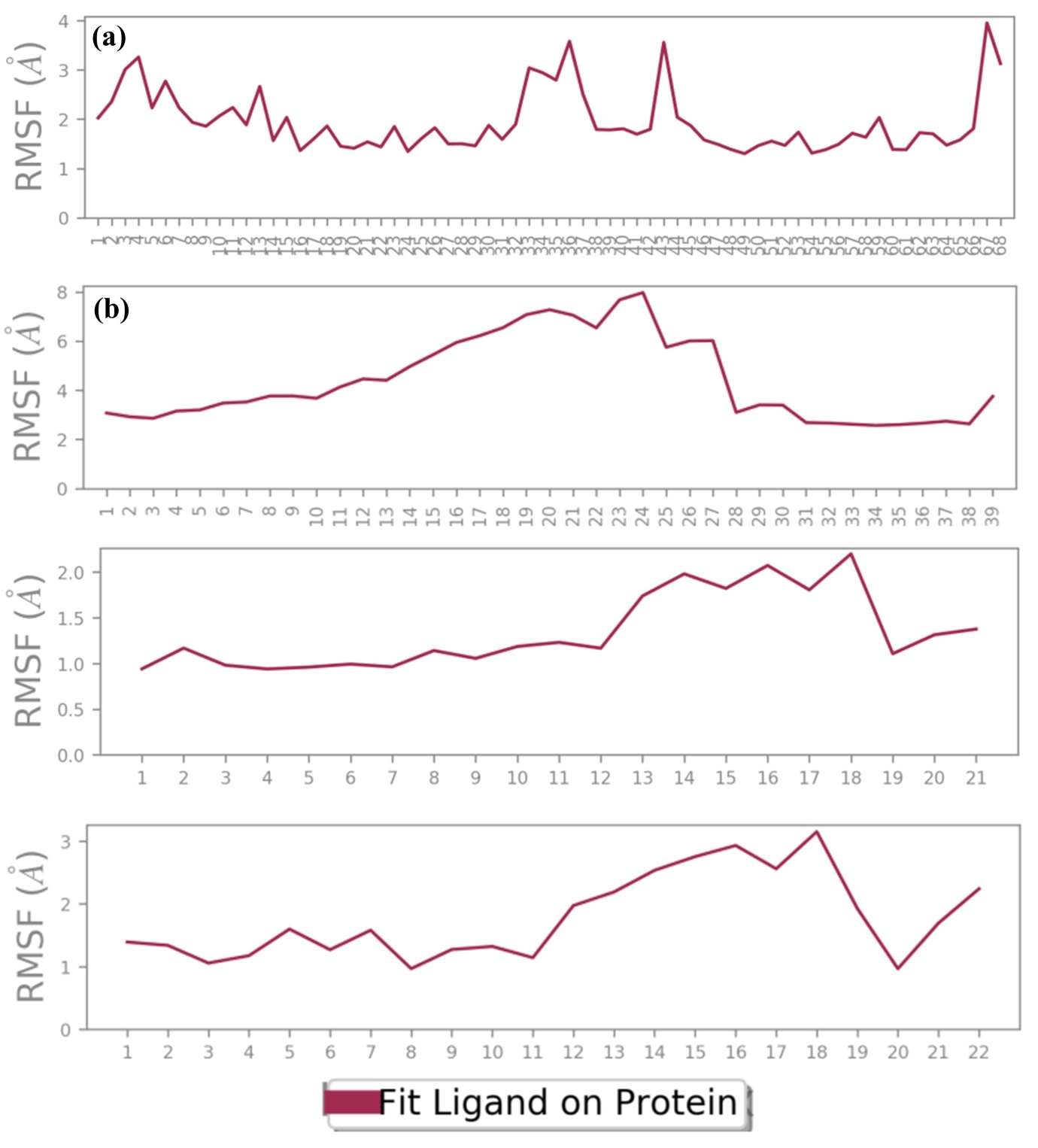


**Figure S6.** RMSF plot generated for the docked phytochemical Compounds, i.e., (a) Unii-CQ2F5O6yiy, (b) Lithospermic acid, (c) Kaempferol and (d) Rhamnocitrin fit in the cysteine proteinase during 250ns molecular dynamics simulation interval.


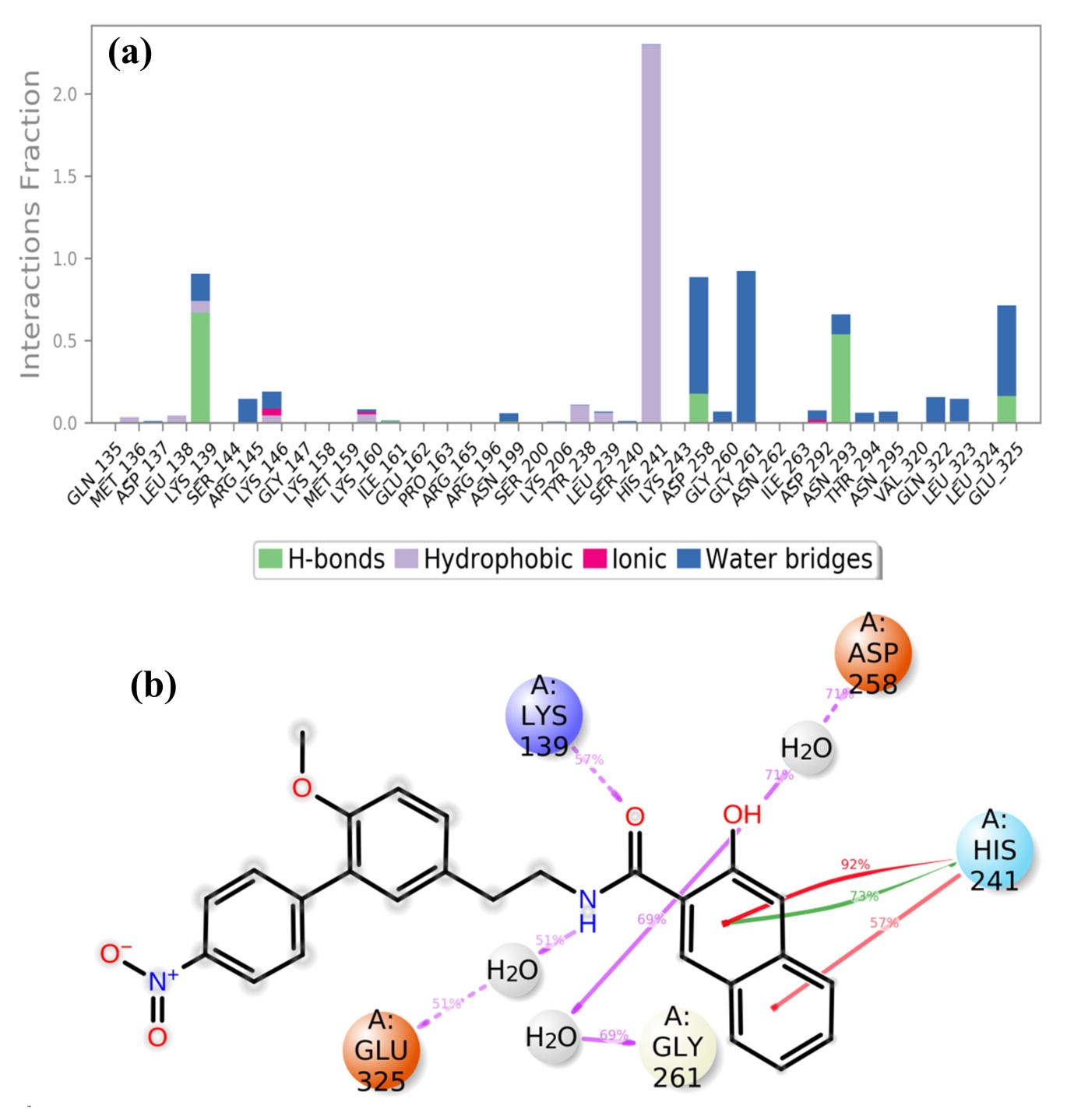


**Figure S7**. (a) Protein-ligand interaction mapping for cysteine proteinase docked with reference ligand TTP-6171 extracted from 250 ns molecular dynamic simulations, (b) a detailed schematic representation of atomic interaction of reference ligand, docked with cysteinessss proteinase. Interactions that occur more than 30.0% of the simulation time in the selected trajectory (0.00 through 250.04 ns), are shown.

**S1.6. Binding free energy analysis**

**Table S4.** Calculated net Binding free energy and energy components values per frame for cysteine proteinase complexes with phytochemical compounds.

| **Energy**  **components** | **Unii-CQ2F5O6yiy** | **Lithospermic acid** | **Kaempferol** | **Rhamnocitrin** | **TTP-6171** |
| --- | --- | --- | --- | --- | --- |
| **∆G Bind** | -53.02±7.9 | -43.36±8.09 | -39.29±5.85 | -38.22±4.2 | -44.06±4.97 |
| **∆G Bind Coulomb** | -27.01±9.11 | -28.94±38.13 | -28.96±6.67 | -19.35±3.9 | -12.88±3.74 |
| **∆G Bind Covalent** | 3.05±2.58 | 2.35±1.84 | 2.67±2.0 | 1.54±1.79 | 2.18±1.84 |
| **∆G Bind Hbond** | -3.13±1.24 | -3.72±0.88 | -2.38±0.57 | -1.95±0.41 | -1.56±0.48 |
| **∆G Bind Lipo** | -14.15±2.06 | -8.06±2.79 | -6.47±0.83 | -8.56±1.33 | -9.47±2.21 |
| **∆G Bind Packing** | 0 | -0.79±0.74 | -3.11±0.57 | -1.93±0.7 | -3.72±0.33 |
| **∆G Bind Solv GB** | 46.75±5.62 | 27.64±35.74 | 27.41±2.71 | 24.42±2.6 | 25.49±3.3 |
| **∆G Bind vdW** | -58.52±4.64 | -31.83±7.07 | -28.45±2.57 | -32.39±3.41 | -44.09±3.6 |
| **Lig Strain Energy** | 8.26±4.02 | 5.3±2.85 | 2.92±2.3 | 1.85±1.73 | 4.15±2.28 |

**
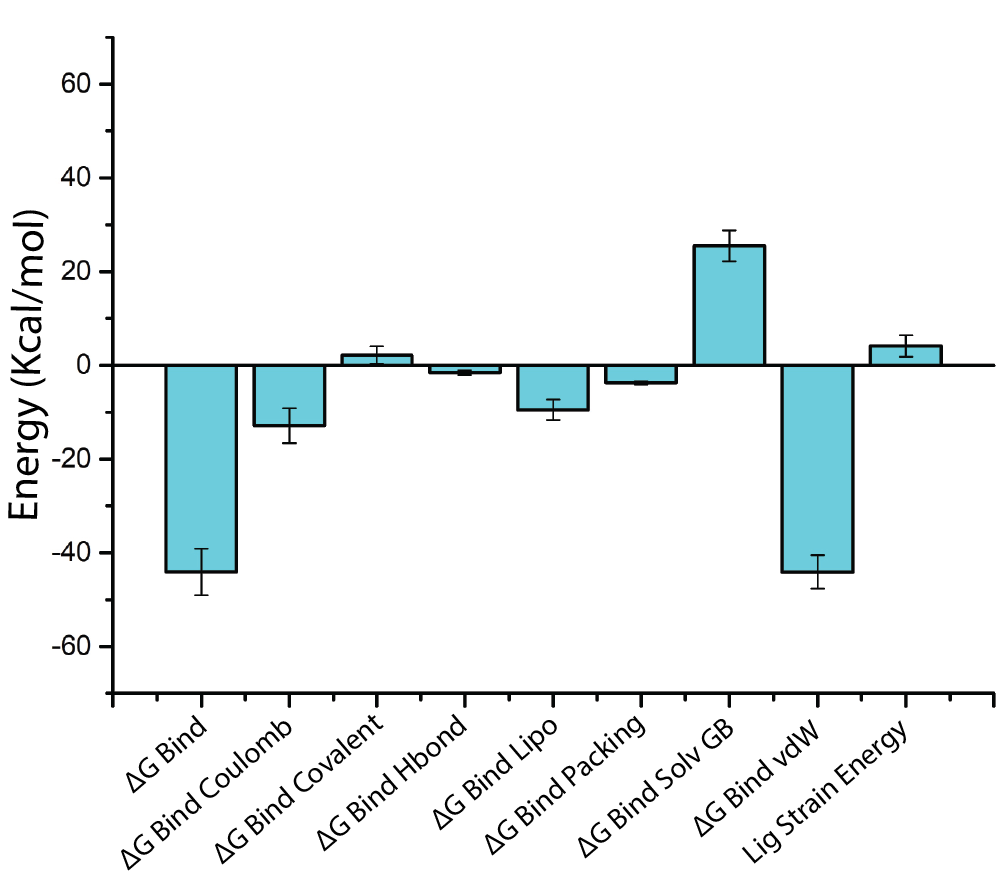
**

**Figure S8.** Calculated net Binding free energy and energy components values per frame for cysteine proteinase complex with TTP-6171 reference molecule.
